# Characterization of Multilayer Coupling Based on Square Complementary Split Ring Resonator for Multiport Device Implementation

**DOI:** 10.3390/mi14010068

**Published:** 2022-12-27

**Authors:** Eduardo Jarauta, Juan Carlos Iriarte, Francisco Falcone

**Affiliations:** 1 Electrical Engineering and Communications Department, Universidad Pública de Navarra, Campus Arrosadía, E-31006 Pamplona, Spain; 2 Institute of Smart Cities, Universidad Pública de Navarra, Campus Arrosadía, E-31006 Pamplona, Spain; 3 Tecnologico de Monterrey, School of Engineering and Sciences, Monterrey 64849, Mexico

**Keywords:** microstrip multilayer, multilayer diplexer, multilayer resonator, complementary split ring resonator

## Abstract

The advent of context-aware environments and related applications demands a high degree of connectivity, with new spectral bands and related radio resource management functionalities in the current 5G bands and foreseen in future 6G wireless communication systems. This, in turn, poses new challenges in the implementation of wireless transceivers and radiating systems, in terms of device integration, miniaturization and element isolation, among others. High-performance miniature devices are presented and studied in this work, aided by metamaterial-inspired complementary resonators. A single particle is used to build a single layer, double layer, double frequency resonators and power dividers. A complete characterization of each equivalent circuit is also analyzed, showing great agreement between analytical circuit models and full-wave electromagnetic simulations. By adding more particles, different diplexers and triplexers in the multi-layer configuration are proposed. The flexibility in the design is the key advantage, as all devices are easily tunable and the output lines can be built in different layers, enabling frequency scalability from RF to millimeter wave ranges. Nevertheless, they are only a sample of all possible combinations of devices that can be designed for integration in future wireless communication systems.

## 1. Introduction

The advances foreseen in the implementation of context-aware environments given by the Internet of Things (IoT) in multiple applications and domains within smart cities and smart regions are by great means supported by wireless communication systems. In this sense, there has been a sustained effort in the implementation of different solutions within the paradigm of heterogeneous wireless networks in order to provide adequate coverage/capacity relations whilst complying with increasing demands in terms of energy consumption reduction and optimization, decrease in size and form factor and more robust response in increasing interference environments, among others [1,2,3,4]. This has catalyzed the development of multiple devices integrated within wireless transceivers as well as in radiating systems. In this sense, metamaterials have been explored for multiple facets of device/system optimization, spanning from device miniaturization, isolation improvement or the proposal of novel radiating solutions, among others [5,6,7,8,9].

In this context, coupling in multilayer microstrip devices was studied deeply during the last century [10]. Many techniques were developed to build all kinds of multi-layer microstrip devices [11]. The singular interest of this study is the design of multilayer microstrip couplers [12,13]. Analysis of slot apertures in multilayer microstrip structures was also deeply studied [14]. The design by coupling through a slot was widely developed and analytically studied [15,16,17]. This knowledge was used in the design of directional couplers in the double substrate layers [18]. The result is directional couplers with similar characteristics to single-layer couplers. The coupling frequency can be also tuned by modifying slot dimensions. A theoretical extension for multilayer structures was also studied, with a practical implementation in a two-layers directional coupler [19]. Other configurations have been used to design multilayer couplers and power dividers in microstrip [20,21], or other approaches [22].

In this paper, slot coupling will be explored by employing Complementary Split Rings Resonators (CSRRs) in order to implement multiple planar technology devices. The key outcomes which are followed in this paper are two. The first one is to reduce the fingerprint with the miniaturization of devices, owing to the inherent subwavelength operation of CSRR elements. The second one, in addition to miniaturization, is the use of multilayer structures which provide flexibility in the design and subsequent integration of the proposed devices, reducing surface footprint as well as enabling multilevel connector settings. CSRRs were presented in 2004 [22], for the first time, as an evolution of Split Ring Resonator (SRR) particles [23] in which axial E-field excitation (e.g., microstrip transmission lines, substrate integrated waveguides, etc.) is adequate when contained in the normal plane [24]. Its properties include its small size and good resonance in its quasi-static resonant frequency. The different types of devices that are presented in this work are outlined in Table 1 and will be described in the different sections.

## 2. Complementary Split Ring Resonator

**Figure 1:**
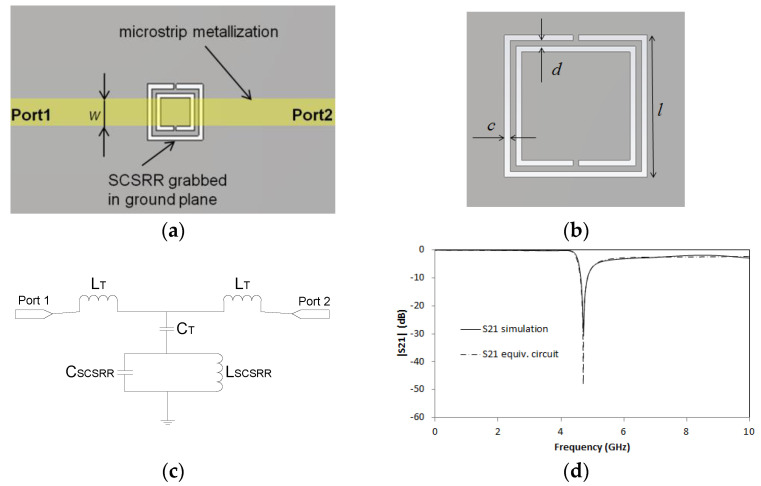
(**a**) Top layout SCSRR. (**b**) SCSRR main dimensions. (**c**) Equivalent circuit model for SCSRR. (**d**) Simulated S21 (solid black line) and simulated S21 for equivalent circuit model (dash-dotted line).

The CSRR’s have been intensively used for all kinds of devices, from filters [25,26,27,28] to couplers [29], and, in recent years, there has been growing interest in using them for sensors [30,31]. Its equivalent circuit, namely SRR’s, was characterized in [32] for the first time. This approach was enhanced in different configurations [33,34] and for different applications [35]. In this letter, a Square Complementary Split Ring Resonator (SCSRR) is used. In this article, the usage of a complementary resonator is considered more convenient than a metallic resonator SRR for two reasons. The excitation of the rings, in the case of the serial SSRR, is much better when it is excited by its electric field. The second motivation is that it also obtained better coupling between the resonator and the output lines on different layers, as we will demonstrate. In Figure 1a, the layout of a microstrip line with an SCSRR etched in the ground plane is displayed. To establish the dimensions of our device, we first set the resonance frequency. From the equations provided in [32], it is understood that the radius of the conventional SRR is resonant at that frequency. In this case, it has been adapted by equating the perimeter of our square resonator with the length of the circumference of the already calculated SRR. The dimensions for the Square Complementary Split Ring Resonator, displayed in Figure 1b, used in the first part of this paper are the following: length of the square side *l* = 5 mm. The thickness of the rings *c* = 0.2 mm. Distance between rings *d* = 0.2 mm. Finally, the microstrip line width *w* = 2.4 mm, which is the needed value to adapt the line to 50 Ω.

The equivalent circuit for the SCSRR contains the same components used to model the CSRR [32]. The inductance L_SCSRR_ and capacitance C_SCSRR_ are calculated by considering the perimeter of the square, the width of the SCSRR (*c*) and the separation between rings (*d*), and by applying the same electromagnetic calculations provided in [32]. The microstrip line is characterized by its T-model [36], with its lumped components *L_T_* and *C_T_*, where *L_T_* and *C_T_* are the per-section inductance and capacitance of the microstrip line. The circuit is painted in Figure 1c.

The circuit has been simulated using the commercial substrate RT Duroid 5880 with εr = 2.2 and substrate thickness *h* = 0.79 mm. This substrate will be used for all the devices throughout this paper. For a microstrip line with the dimensions detailed in the previous paragraph, the values of the elements of the equivalent circuit are L_T_ = 1.16 nH, C_T_ = 0.296 pF, C_SCSRR_ = 0.38 pF and L_SCSRR_ = 1.69 nH. The electromagnetic simulations in this paper were made with commercial software CST Microwave Studio^TM^, using both methods transient solver and frequency domain solver in order to validate the results. Simulation results and simulation of the equivalent circuit model are displayed in Figure 1d.

A good agreement between full wave simulation and the equivalent circuit model is displayed in the results. In both cases, the resonant frequency obtained at *f_0_* = 4.71 GHz validates the approach of the square resonators that will be used in the paper. It is important to note that this resonator is tunable by modifying resonator dimensions as per the equations in [32].

## 3. Resonators

In this section, different topologies of resonators using the Square Split Ring Resonator (SSRR), or its complementary (SCSRR), will be analyzed and characterized with their equivalent circuit. The previous studies [32] and [34], will serve as a starting point in the analysis of the equivalent circuits presented in this paper.

A serial configuration of SRR in a single-layer microstrip line was presented [37]. This serial configuration is of particular interest because the SRR is excited by the electric field parallel to the resonator. This is due to the bi-anisotropy property of a single SRR, as it was described in [38]. As a consequence, one of the main features is that a high Q is obtained as the resonance occurs in the sub-lambda frequency of the ring, with low insertion losses.

**Figure 2:**
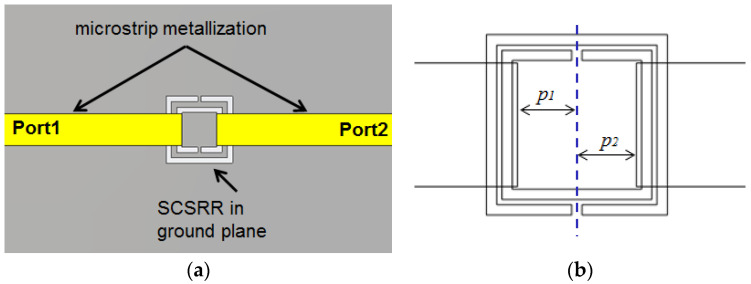
(**a**) Layout of the SCSRR resonator. (**b**) Detailed parameters *p1* and *p2* to build the open stub.

The first device proposed in this article is a variant of the above-mentioned particle. The SRR used in the original paper will be replaced with a Square Complementary Split Ring Resonator (SCSRR) in the same serial configuration. The microstrip input line will be cut-off just above SCSRR. The output microstrip line is placed on the opposite side of the SCSRR, as shown in Figure 2a.

A relevant parameter in the design of the circuit is the distance between the geometric center of the SCSRR and the beginning of the input microstrip line (*p_1_*) and output microstrip line (*p_2_*). The values are represented in Figure 2b. The open microstrip line builds an open stub in both the input and output lines to make the lines adapted.

**Figure 3:**
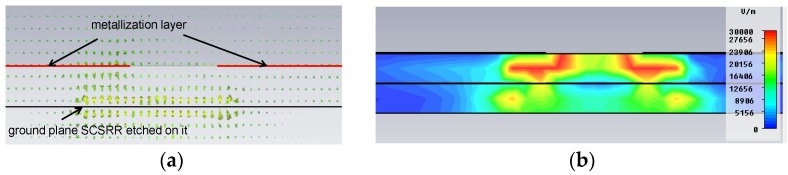
E-field SCSRR excitation. (**a**) Vector plot. (**b**) Absolute E-field values.

In this configuration, the SCSRR is excited in its quasi-static frequency by the axial electric field of a 50 Ω microstrip line. The signal is coupled again from the SCSRR to the output 50 Ω microstrip line. Figure 3a plots the vector E-field representation in a front view of the previous device. On the left side, the typical microstrip E-field distribution is appreciated. At the end of the microstrip line (a red line labeled as the metallization layer), a strong normal E-field is achieved on the ground plane. The main concentration of the E-field is observed in the region where SCSRR is etched on the ground plane. Finally, on the right side, it can be observed how the E-field is coupled with the output line again. In Figure 3b, the absolute values of the E-field are painted, which confirms all that was explained above, as the highest values were re-obtained in the regions between the SCSRR and the end of the microstrip lines.

**Figure 4:**
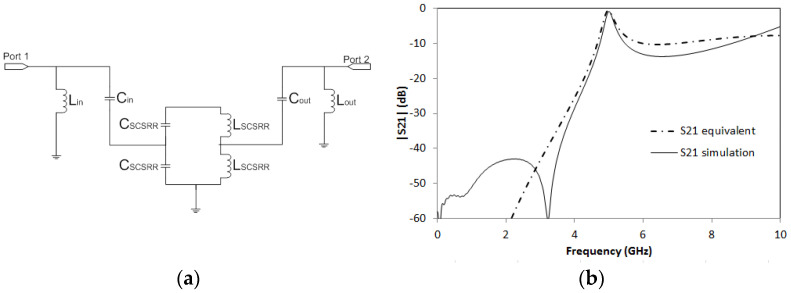
(**a**) Equivalent circuit model for SCSRR Micro-Resonator. (**b**) Full wave simulation results for S21 (solid black line) compared to the simulated S21 obtained with the equivalent circuit model (dash-dotted line).

The resonator in this device is characterized by its main LC components [32]. The energy coupled from the input line to the SCSRR is modeled as a capacitor, *C_in_*, and it is calculated as a parallel plate between both metallization layers, microstrip feed line and metallization where SCSRR is placed. The width would be in this case the width of the line (*w*). The end of the input line as a single open stub, *L_in_* [36], the output line, is characterized again with a capacitor, coupling from the resonator to the output, *C_out_* and again an inductor represents the open stub, *L_out_*, in the output microstrip line. The complete circuit is displayed in Figure 4a.

Using the aforementioned substrate and with the dimensions of the ring resonator defined previously, the values of the components are *L_in_* = 1.16 nH. *C_in_* = 0.296 pF, *C_SCSRR_* = 0.81 pF, *L_SCSRR_* = 0.87 nH. *C_out_* = 0.296 pF and *L_out_* = 1.16 nH. In Figure 4b, the simulation results are compared with the equivalent circuit model presented. Great resonance is achieved with a value of S21 = −0.92 dB at the resonant frequency *f_0_* = 5.015 GHz.

**Figure 5:**
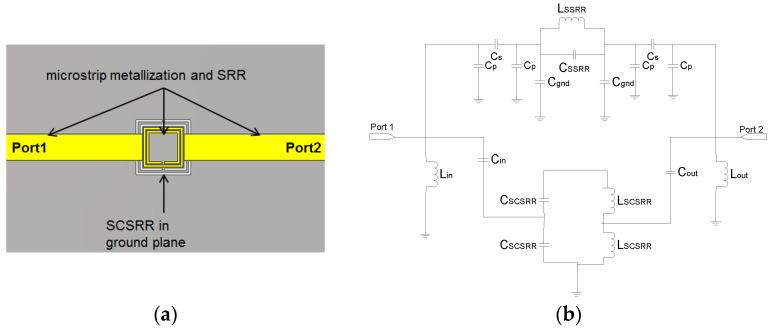

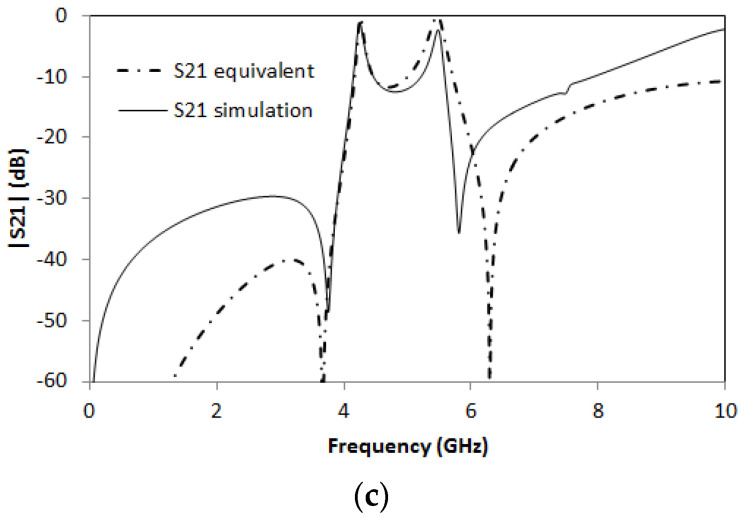
Double frequency resonator. (**a**) Top view overlapped layers. (**b**) Equivalent circuit model. (**c**) Simulated S21 (solid black line) and simulated S21 for equivalent circuit model (dash-dotted line).

Taking advantage of the excitation properties of both SSRR and SCSSR a novel device is presented in this paper. It is made in the combination of a square SSR micro-resonator already presented in [37] and the SCSRR resonator just presented above. Both resonators share input and output microstrip lines as it is represented in overlapped layers layout of Figure 5a. The main advantage of this device is that it obtained a tunable double-frequency resonator [32] and a really small single particle size. Only one design requirement is needed, namely the SSRR length side must be smaller than the SCSRR one. The equivalent circuit for this device is straightforward. It is characterized by a parallel design of the equivalent circuit model of the SRR micro-resonator [37] and the circuit presented for the SCSRR resonator validated in Figure 4. The equivalent circuit for the double-frequency resonator is drawn in Figure 5b.

The Square Split Ring Resonator on the top layer has a side length *l_1_* = 3.4 mm, width of the rings *c_1_* = 0.2 mm, separation between rings *d_1_* = 0.1 mm and distance between microstrip input and output lines *s_1_* = 0.1 mm. The SCSRR has a side length *l_2_* = 5 mm, width of the rings *c_2_* = 0.2 mm, and separation between rings *d_2_* = 0.1 mm, considering the substrate, as in the previous case. The values for the equivalent circuit, considering the dimensions of the rings, and substrate described above are, firstly, the upper part which represents the Square Split Ring Resonator (SSRR): *C_s_* = 0.091 pF, *C_p_* = 0.0093 pF, C_gnd_ = 0.006 pF, *L_SSRR_* = 7.1 nH, and *C_SSRR_* = 0.062 pF. The lower part represents the SCSRR: *L_in_* = 1.1 nH, *C_in_* = 0.24 pF. *C_SCSRR_* = 1.12 pF and *L_SCSRR_* = 1 nH. *C_out_* = 0.24 pF, and *L_out_* = 1.1 nH. Simulation and equivalent circuit response are displayed in Figure 5c.

The agreement between the simulation and the equivalent circuit demonstrates the validity of the analysis proposed. A moderate deviation in the zero frequency is observed that can be caused by surface wave excitation or coupling derived from the presence of elements such as the corners of square resonators, which are not feasible to include in the model. Two resonances are achieved, the first one at *f_0_* = 4.24 GHz, corresponding to the CSRR resonance. The second one at *f_1_* = 5.51 GHz is the resonant frequency of the SSRR. The results show a high performance for both resonances. It also makes this particle a promising alternative in the design process for multiple applications. In a single particle, we observe both resonances, which improve the response of previous solutions [39]. It also reduces the effective area used to print the device.

**Figure 6:**
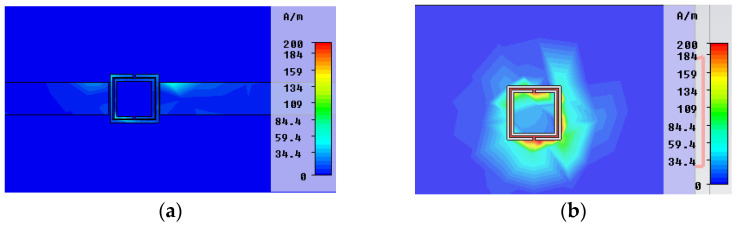

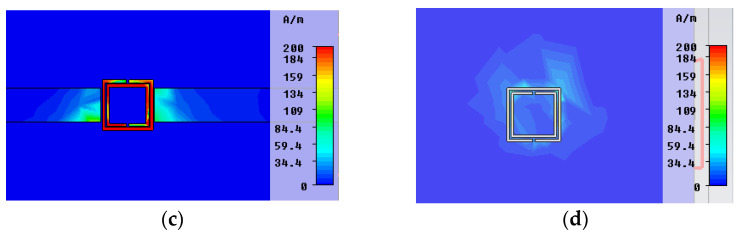
Surface current distribution. (**a**) Surface current in upper metallization layer at *f_0_* = 4.24 GHz. (**b**) Surface current in bottom metallization layer at *f_0_* = 4.24 GHz. (**c**) Surface current in upper metallization layer at *f_1_* = 5.51 GHz. (**d**) Surface current in bottom metallization layer at *f_1_* = 5.51 GHz.

In the next figure, the surface current distribution in the top metallization layer, and bottom metallization layer, in both resonant frequencies, are depicted. In Figure 6a,b, it can be seen that in the first resonant frequency *f_0_* = 4.24 GHz, only the SCSRR is excited, while SSRR is almost transparent. In the second resonant frequency *f_1_* = 5.51 GHz, most of the surface currents are cumulated around the SSRR while SCSRR has no influence on it.

**Figure 7:**
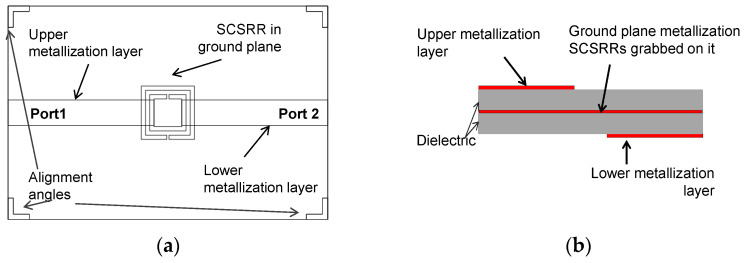
(**a**) Overlapped top view serial 2-layer resonator. (**b**) Schematic front view layer distribution.

A new device, based on a two microstrip layers composite, is proposed in this study. It is based on the SCSRR serial resonator presented in the previous chapter. In this case, the upper metallization layer contains a 50 Ω source line. The next metallization layer is the ground plane, which is shared by both microstrip lines. In this metallization layer, an SCSRR is etched. Finally, in the bottom layer, a new 50 Ω line is placed, acting as the output port. The overlapped layout is displayed in Figure 7. The design is made by assembling two microstrip plates. Both use the same dielectric substrate defined in Section 2. One of the plates has copper metallization on both sides. In one of them, the input line is grabbed. In the second metallization layer, which represents the ground plane, the SCSRR was etched. The second substrate plate has copper metallization only on one edge. In this metallization, the output microstrip line was drawn. It is represented as a lower metallization layer. The schematic front view with the layer distribution is depicted in Figure 7b.

**Figure 8:**
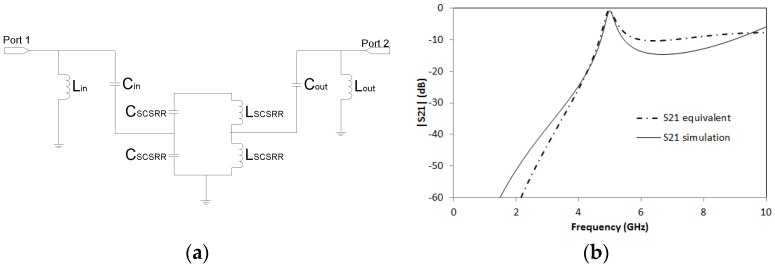
(**a**) Equivalent circuit model 2-layer resonator previously depicted. (**b**) Simulated S21 (solid black line) and simulated S21 for equivalent circuit model (dash-dotted line).

For this device, the proposed equivalent circuit is depicted in Figure 8a. An input microstrip line is coupled to the SCSRR and from there, the currents are also coupled to the output line. Two inductances *L_in_* and *L_out_* represent the stubs made by the opened microstrip lines. *L_in_* = 1.16 nH. *C_in_* = 0.296 pF. *C_SCSRR_* = 0.81 pF and *L_SCSRR_* = 0.87 nH. *C_out_* = 0.296 pF and *L_out_* = 1.16 nH. The simulation results as well as the calculation of the equivalent circuit are plotted in Figure 8b. This resonator obtains a peak in the magnitude of the S21 value of −0.83 dB at its resonant frequency *f_0_* = 5 GHz. This result is comparable to other multilayer resonators [40,41] with the advantage of low insertion loss, small size, and flexibility.

Good agreement is again achieved between the simulation and equivalent circuit at the working frequencies of the resonator. There is a small deviation in lower frequencies and also in higher frequencies that could be caused by surface wave coupling or higher modes that were not considered. It is important to highlight that insertion losses are also comparable to the previously presented device in which output lines are placed in the same layer, which provides additional flexibility.

**Figure 9:**
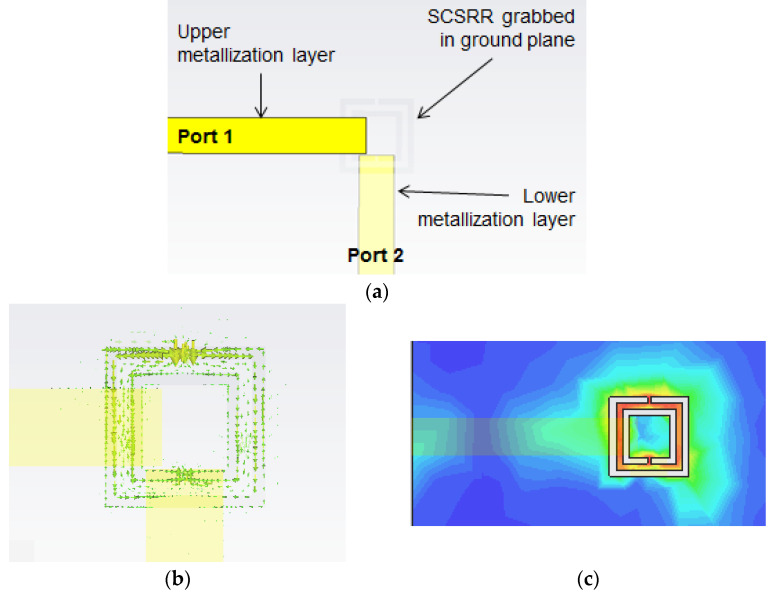
(**a**) Top view perpendicular two-layer resonator and surface current distribution. (**b**) Vector plot of surface current. (**c**) Magnitude plot within the CSRR region.

The last resonator presented in this section is also a variant of the previous devices. The layout is shown in the next picture. The main difference in this device’s design is that the output is placed perpendicular to the input microstrip (in the bottom layer again). The overlapped top layout is shown in Figure 9a.

As demonstrated in [24], most surface currents are accumulated in gaps between rings in the CSRR. In Figure 9b,c, the surface current distribution at the resonant frequency is displayed. The beginning of the output line to Port 2 is selected just below the gap placed in the internal ring. In this device, the dimensions of the SCSRR are the same as in previous devices. The main difference is the values for adapting parameters *p_1_* = 0.7 mm and *p_2_* = 1.3 mm.

**Figure 10:**
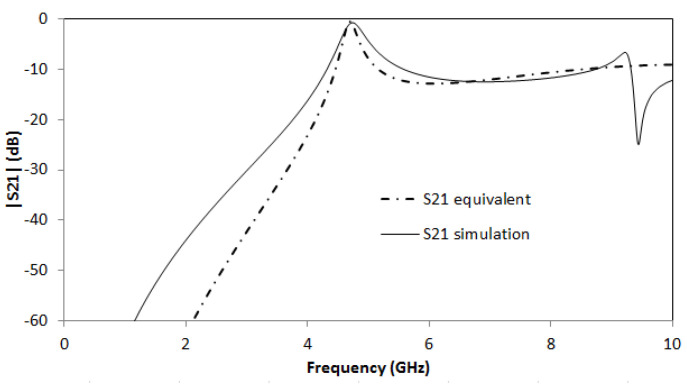
Simulated S21 (solid black line) and simulated S21 for equivalent circuit model (dash-dotted line).

In this case, the components of the equivalent circuit are the same as the ones defined for the SCSSR resonator (Figure 4a). In this case, the input inductance *L_in_* has a value of 1.16 nH. The capacitor coupled to the ground plane *C_in_* has a value of 0.296 pF. The values for *C_SCSRR_* = 0.81 pF and the *L_SCSRR_* = 0.87 nH. Finally, the coupling from the resonator to the output line is characterized by *C_out_* = 0.137 pF. The stub in the output line is characterized by inductance *L_out_* = 1.16 nH. Figure 10 displays the simulation and equivalent circuit results of the previous device. Insertion loss at resonant frequency *f_0_* = 4.74 GHz. has a value S21 = 0.71 dB. A similar deviation is observed at lower frequencies, owing to the effects previously stated. By comparing these results with the ones obtained for devices in Figure 4b and Figure 8b, comparable performance is achieved, giving additional flexibility in the design of new devices.

To summarize, the results in this chapter (see Table 2) collect key figures for the presented devices compared with previous studies on multi-layer microstrip resonators.

The results in the table summarize the benefits of this design. For a similar size, lower insertion losses are obtained in an effectively smaller area.

## 4. Power Dividers

**Figure 11:**
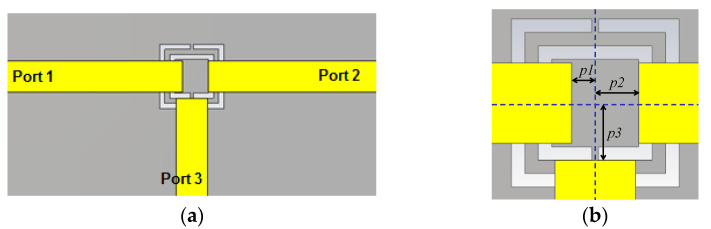
(**a**) Top view SCSRR single layer power divider. (**b**) Detail of microstrip lines position over the SCSRR.

SRRs have already been used in the design of power dividers. In the last years, some implementations based on Substrate Integrated Waveguide (SIW) [42,43] have been proposed. The balun power divider [44], the planar technology of the Bailey power divider [45] and the Wilkinson power dividers [46] have been implemented using SRR or CSRR as the resonant element. In this work, a comparison with different implementations of multilayer power dividers will be offered. The idea of building a multilayer power divider based on coupling energy through a slot was successfully implemented [47]. Composite right-handed and left-handed power dividers have also been used in the design of multilayer power dividers [48]. In this section, a new design for multilayer power dividers is presented. The idea is born from the knowledge of the surface current accumulation in the gaps of SCSRR at its resonant frequency. The first power divider can be seen in Figure 11. The base is the resonator proposed in Figure 2a. This resonator is converted to a power divider by adding an output microstrip line, which is perpendicular to the input-output axis, and the line starts over the SCSRR.

The end of the input line and the beginning of the output lines, to Ports 2 and 3, have been designed following an optimization process to obtain an adapted stub to the 50 Ω microstrip lines. To achieve the distances from the center of the ring to the beginning of the lines, labeled as *p1*, *p2* and *p3* were optimized (Figure 11b). The distance between the end of the input line and the center of the device is *p1* = 0.7 mm. The distance between the device’s center to the beginning of the output line to Port 2 is *p2* = 1.3 mm, and the distance between the center and the output line to Port 3 is *p3* = 1.7 mm. 

**Figure 12:**
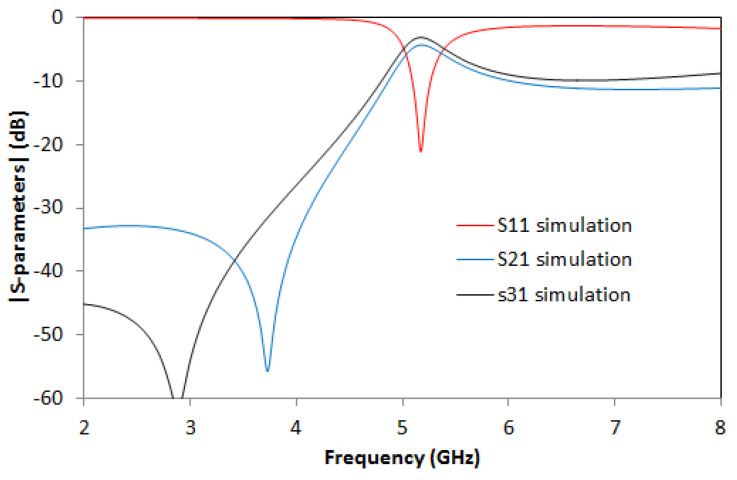
Simulated S21 (solid blue line), simulated S31 (solid black line), and simulated S11 (solid red line).

The results from the simulation are shown in Figure 12. Values of S21= −4.69 dB and S31 = −4.03 dB are obtained at the resonant frequency of *f_0_* = 5 GHz.

**Figure 13:**
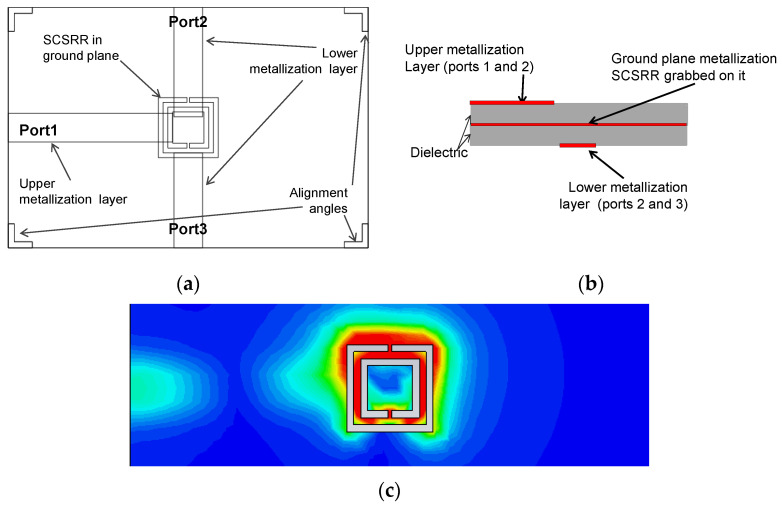
(**a**) Top view layer distribution of a 2-layer power divider. (**b**) Front view layer distribution. (**c**) Surface current distribution in SCSRR metallization layer at the resonant frequency.

The next device presented is a variant of the previous one. The input line is again a microstrip line that ends on the top of an SCSRR which is grabbed in the metallization layer. In a new microstrip line placed just below the metallization layer, two output microstrip lines are built, and they share the ground plane with the input line. Each output line starts just over each gap of the SCSRR, with a small adjustment in the position at the end of the line to achieve the best adaptation in both output lines, which are parameters *p2* and *p3,* as previously explained. Finally, an angle is drawn on each corner of the device. This is drawn to facilitate the assembly of the two plates since accuracy is critical to obtain the best adaptation, which means the best response to the outputs. In Figure 13c, the surface current distribution at the resonant frequency is depicted. Most of the currents are cumulated in the gaps of the SCSRR, being the optimal position to couple energy to the output lines.

**Figure 14:**
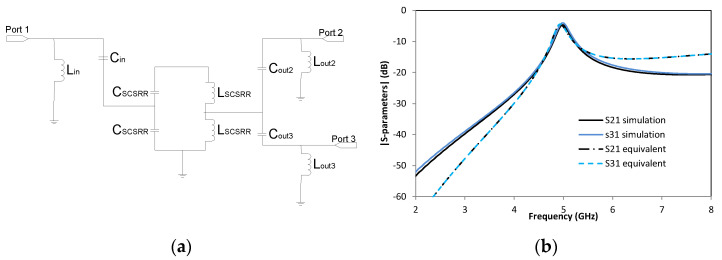
(**a**) Equivalent circuit model 2-layer power divider. (**b**) Simulated S21 (dashed black line) and simulated S31 (dashed blue line). Equivalent circuit S21 (dashed-dotted black line) and equivalent circuit S31 (dashed blue line).

The equivalent circuit of this device is the same for the input, which means a microstrip entry is coupled to the SCSRR through a coupler *C_in_*. The open stub is emulated with inductance *L_in_*. The output differs from devices in the previous section because this is a power divider, but the idea is the same. The currents are coupled from the SCSRR to the output line of Port 2 through coupler *C_out2_* and the adaptation is achieved with the open stub of the line represented as inductance *L_out2_* (which is the same for Port 3). Coupling is characterized in *C_out3_* and adaptation with *L_out3_* inductance. The complete layout is plotted in Figure 14a.

With the dimensions of the prototype proposed, the values for the elements in the equivalent circuit are *L_in_* = 1.16 nH and *C_in_* = 0.296 pF. The value of SCSCRR elements is the same, namely *C_SCSRR_* = 0.81 pF and *L_SCSRR_* = 0.87 nH. The output components have the following values: *C_out2_* = 0.19 pF, *L_out2_* = 1.16 nH, *C_out3_* = 0.19 pF and *L_out3_* = 1.16 nH. The S-parameter response to the circuit is shown in Figure 14b. We observe a value of S21= −4.69 dB and S31 = −4.03 dB at the resonant frequency *f_0_* = 5 GHz.

The results show a high-efficiency power divider, having output lines in a different layer than the input line, using a single particle SCSRR. This makes the device competitive because of its small size and good performance. It is also important that a completely symmetrical response is obtained in both output ports. As the equivalent circuit is completely symmetrical, the S21 and S31 lines for the equivalent circuit are overlapped because of that a different style is chosen for each data series. The equivalent circuit matches the simulation perfectly in working frequencies and shows a short deviation in lower and higher frequencies that can probably be caused by some coupling that is not considered because of the proximity of the input and output lines.

**Figure 15:**
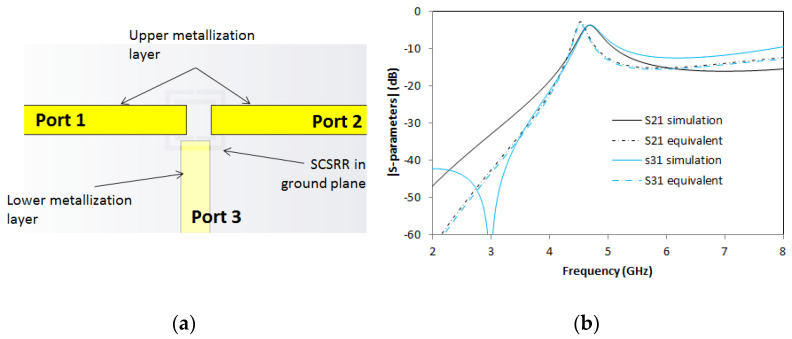
(**a**) Top view overlapped layer distribution of a 2-layer power divider. (**b**) Simulated S21 (solid black line) and simulated S31 (solid blue line). Equivalent circuit S21 (dashed-dotted black line) and equivalent circuit S31 (dashed-dotted blue line).

A variant of the previous power divider is presented now. It is built as a combination of the presented devices. This time, the power divider outputs are designed in different layers. Output in Port 2 is placed in the same layer as the input. Output in Port 3 is in a layer below the ground plane, following the same approach as the previous device. A layered top view is drawn in Figure 15a.

In Figure 15b, the S-parameters are shown. At the resonant frequency *f_0_* = 4.7 GHz, insertion losses are S21 = −3.71 dB and S31 = −3.69 dB, giving an efficiency of this divider of 91%. In this case, S21 and S31 do not have a symmetrical response. In this case, a moderate deviation in the resonant frequencies is observed, owing to surface wave coupling, excitation of higher order modes and/or coupling effects between input/output lines that were not considered in the equivalent circuit.

**Figure 16:**
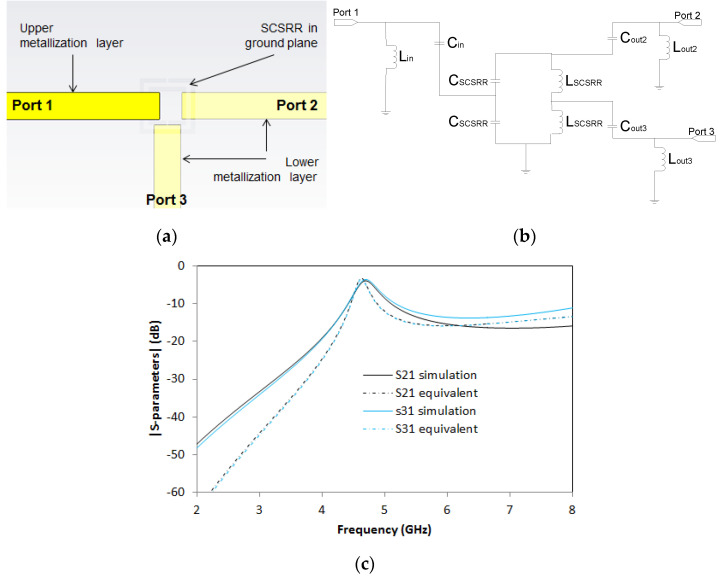
(**a**) Top view layer distribution 2-layer power divider, with two outputs in second layer. (**b**) Equivalent circuit model 2-layer power divider. (**c**) Simulated S21 (solid black line) and simulated S31 (solid blue line). Equivalent circuit S21 (dashed-dotted black line) and equivalent circuit S31 (dashed-dotted blue line).

A new variant is proposed in Figure 16. It is quite similar to the previous one. This time, the output in Port 2 is also placed in the layer below the ground plane, as it is placed in Port 3. The top layout is displayed in Figure 16a. For this device, the dimensions are exactly the same as the previous one. With the dimensions of the prototype designed, the values for the elements in the equivalent circuit are *L_in_* = 1.16 nH, *C_in_* = 0.296 pF, and the value of the SCSCRR elements are the same, namely *C_SCSRR_* = 0.81 pF and *L_SCSRR_* = 0.87 nH. Output components have the following values: *C_out2_* = 0.137 pF, *L_out2_* = 1.16 nH, *C_out3_* = 0.296 pF and *L_out3_* = 1.16 nH. A picture of the device is provided in Figure 16b.

The full wave simulation results, and equivalent circuit results, can be seen in Figure 16c. For this device, the resonant frequency is obtained at *f_0_* = 4.7 GHz, with S21 = −3.89 dB and S31 = −3.54 db. There are two facts to highlight concerning the output. Complete symmetry in S21 and S31 is achieved for this device. The second important point is that the S21 response is quite different from the previous device’s response, although the top layout is the same. 

To conclude this section, Table 3 shows a comparison between the new power dividers presented in this article and others with a similar design. The criteria for selecting the devices for the comparison are multi-layer devices through a slot in the ground plane or multi-layer power dividers based on ring resonators.

Two results can be highlighted. The first one is that in the proposed design in Figure 15, great efficiency is obtained with similar dimensions as the comparative ones. The second one, which is more relevant from the point of view of the design method, is that it obtained a great efficiency power divider where output lines are placed in different layers.

## 5. Duplexers and Triplexers

As summarized in Table 1, the latest set of devices presented in the article is based on the design of multilayer duplexers and triplexers. Microstrip diplexers have been designed in multiple ways by using composite RH/LH media [49,50]. The usage of resonant particles to achieve great performance duplexers is also common [51,52,53]. There have been also proposed analytical models for multi-stage resonator diplexers [54,55]. This study will focus on the comparison of duplexers in planar technologies such as [56], but it will also share characteristics of multilayer [57] or left/right-handed composites [58].

Again, the initial model is the serial resonator, sometimes in the upper layer and sometimes in the ground plane. It provides the first resonance to the device. The second resonance will be obtained by adding a second SCSRR on the ground plane. A new output line to Port 3 will be placed in the upper or lower layer. Some of the possible combinations will be exposed here.

**Figure 17:**
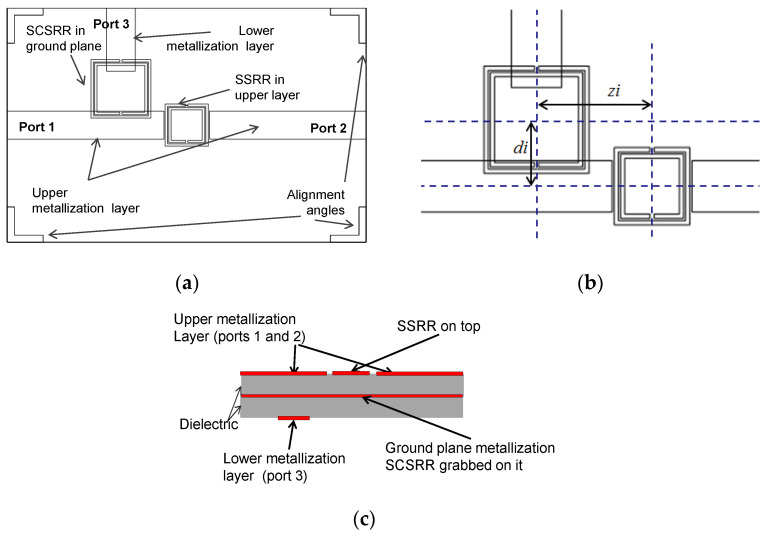
(**a**) Top view overlapped layer distribution of a 2-layer duplexer. (**b**) Relevant dimensions for the second resonator’s position. (**c**) Schematic representation of front view layer distribution.

The first device is a duplexer made with the combination of one SSRR in the serial configuration on the top layer, and one SCSRR grabbed in the metallization layer (see Figure 17a). Output microstrip line to Port 2 is also on the top layer. Output microstrip line to Port 3 is placed in the lower metallization layer (see Figure 17c).

The dimensions of the SSRR placed in the upper layer are square side length *l* = 3.6 mm, the width of rings *c* = 0.2 mm, and separation between rings *d* = 0.1 mm. For the SCSRR placed in the ground plane, dimensions are square side length *l* = 5 mm, the width of rings *c* = 0.2 mm, and separation between rings *d* = 0.1 mm. Finally, the distance between the center of the ring till the beginning of the output line to Port 3, labeled in the previous section as *p3* = 2.7 mm. The distance between the centers of both resonators is labeled as *zi*. The only requirement is that no coupling between rings exists. The second parameter is the displacement of the second resonator from the input microstrip line *di*. Both are shown in Figure 17b.

For this device, the separation between centers is *zi* = 5.5 mm and the displacement of SCSRR from the center of the input line *di* = 3.1 mm. The equivalent circuit of diplexer build with one SSRR and one SCSRR is designed applying the knowledge acquired in previous sections. The upper microstrip layer with an SSRR is modeled with the initial inductance (*L_in_up_*) and capacitance (*Cin_up*) per unit length. The coupling between the line and the SSRR is characterized by a π-model, with the components *Cp* and *Cs*. The SSRR is modeled with the LC tank *L_SSRR_* and *C_SSRR_*. The coupling between the resonator and the ground plane is established with a capacitor (*C_gnd_*). The SSRR is coupled to the output line again characterized as a π-model, and, finally, the output line is modeled with the inductance (*L_out_up_*) and capacitance (*C_out_up_*) per unit length. 

**Figure 18:**
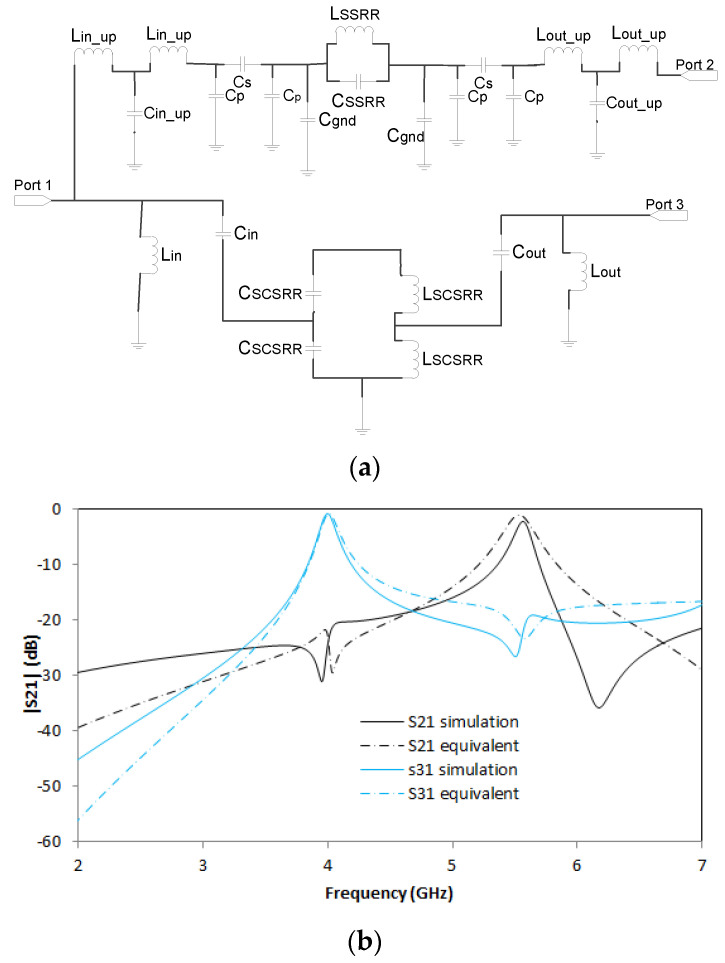
(**a**) Equivalent circuit of diplexer with SSRR on top and SCSRR on ground plane. (**b**) Simulated S21 (dashed black line) and simulated S31 (dashed blue line). Equivalent circuit S21 (dashed-dotted black line) and equivalent circuit S31 (dashed-dotted blue line).

The bottom part is designed as follows: The microstrip line is adapted to the bottom ground plane with inductance (*L_in_*). The line is coupled to the resonator with a capacitance *C_in_*. The complete schema is depicted in Figure 18a.

The values of the equivalent circuit for the diplexer above are, for the upper part: *L_in_up_* = 0.01 nH, *C_in_up_* = 0.04 pF, *Cp* = 0.0093 pF, *Cs* = 0.091 pF, *C_gnd_* = 0.006 pF, *L_SSRR_* = 7.1 nH, *C_SSRR_* = 0.062 pF, *L_out_up_* = 0.01 nH and *C_in_up_* = 0.04 pF. For the lower part the values are *L_in_* = 2.2 nH, *C_in_* = 0.24 pf, *C_SCSRR_* = 1.08 pF, *L_SCSRR_* =1.19 nH, *C_out_* = 0.24 pF, and *L_out_* = 1.6 nH. To calculate *L_SCSRR_* and *C_SCSRR_* the perimeter of the inner square is used. It is also used to obtain the radius of the equivalent circular complementary split ring resonator, and, in this way, to obtain the LC tank values. Finally, both LC components were replaced by two capacitors and two inductors in series. Thus, the value of each *L_SCSRR_* is half of the inductor L calculated in [32]. The value of each *C_SCSRR_* is double the capacitor (C) because it has a built-in serial configuration.

The simulation and measurement results are shown in Figure 18b. For this device, S21 = −2.2 dB is obtained at resonant frequency *f_0_* = 5.57 GHz and S31 = 0.79 dB is observed in *f_1_* = 4 GHz and return losses greater than 20 dB. There is an agreement between the simulation and equivalent circuit models. Deviations in the frequency response are observed in higher frequencies in S21. Although they are in different layers, both SSRR and SCSRR are quite close to each other and it could cause any coupling neglected in our model.

**Figure 19:**
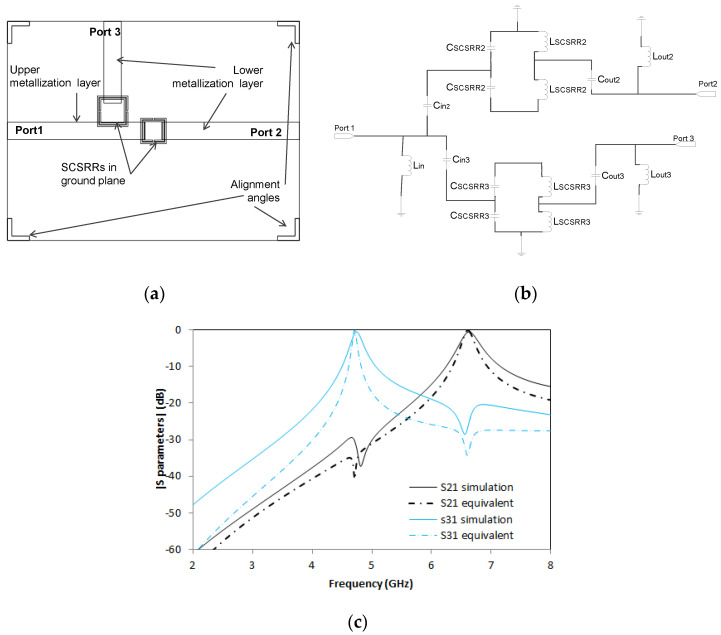
(**a**) Top view overlapped layer distribution of a 2-layer duplexer. (**b**) Equivalent circuit of diplexer made with two SCSRR on ground plane. (**c**) Simulated S21 (dashed black line) and simulated S31 (dashed blue line). Equivalent circuit S21 (dashed-dotted black line) and equivalent circuit S31 (dashed-dotted blue line).

The next device is a small modification of the previous one. In this case, both resonators are placed on the ground plane (see Figure 19a). The dimensions for the smaller SCSRR are square side length *l* = 3.5 mm, the width of rings *c* = 0.2 mm, and the separation between rings *d* = 0.1 mm. Dimensions for the big SCSRR are square side length *l_2_* = 4.2 mm, the width of the rings *c* = 0.2 mm, and the separation between rings *d* = 0.1 mm. The distance between the end of the input line and the center of the device is *p1* = 1.9 mm. The distance between the device’s center to the beginning output line to Port 2 is *p2* = 2.1 mm, and the distance between the center and output line to Port 3 is *p3* = 1.9 mm. Finally, the displacement dimensions for the bigger SCSRR are *di* = 2.7 mm and *zi* = 5.6 mm.

The equivalent circuit for this device consists of two stages of resonators coupled to a microstrip line. The basis of this equivalent circuit is already represented in Figure 19b. The end of the input line is modeled as an inductor Lin, and the coupling between the line and resonators is *C_in2_*, *C_in3_*. The resonant particles for each SCSRR are labeled as *L_SCSRR2_*, *C_SCSRR2_* and *L_SCSRR3_*, *C_SCSRR3_*. From the resonators, the energy is coupled to output lines, designed as *C_out2_* and *C_out3_*, and finally, the adaptation of the output line with the resonator is represented as an open stub called *L_out2_* and *L_out3_*, respectively, for each output line.

The values of the equivalent circuit above, for the circuit dimensions already enumerated are *L_in_* = 3.8 nH, *C_in2_* = *C_in3_* = 0.102 pF, *C_SCSRR2_* = 0.69 pF, *L_SCSRR2_* = 0.74 nH, *C_SCSRR3_* = 0.99 pF, *L_SCSRR3_* = 1.06 nH, *C_out2_* = *C_out3_* = 0.102 pF, and *L_out2_* = *L_out3_* = 5.3 nH.

Simulation and measurement are shown in Figure 19c, exhibiting strong responses in both resonances. In *f_0_* = 6.63 GHz, the insertion loss is S21 = −0.52 dB and the second resonant frequency to the Port 3, *f_1_* = 4.74 GHz with insertion loss value S31 = −0.53 dB. An improvement in S21 insertion loss in comparison to the same device built with SSRR can be seen. Excellent agreement is obtained between the simulation and the equivalent circuit.

**Figure 20:**
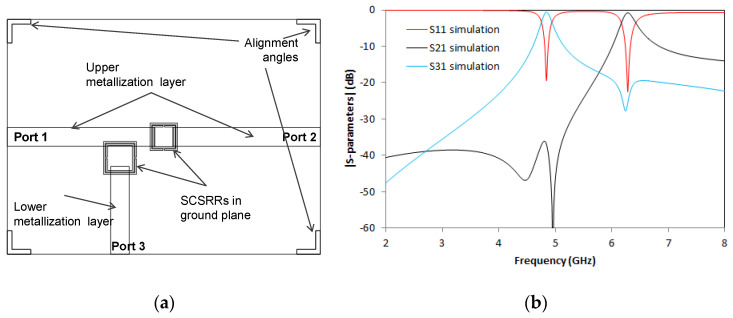
(**a**) Top view overlapped layer distribution of a 2-layer duplexer. (**b**) Simulated S11 (dashed red line), simulated S21 (dashed black line), and simulated S31 (dashed blue line).

Continuing with duplexers, on this new device, output ports are designed in different layers. The output microstrip line to Port 2 is built in the upper metallization layer, while the output microstrip line to Port 3 is in the lower metallization layer, as shown in Figure 20a. All the dimensions on this device are exactly the same as the previous one. As previously stated, the only difference is the location of the output microstrip line to Port 2.

The output for this device is shown in Figure 20b. S21 peak is obtained at *f_0_* = 6.34 GHz and a value of −0.71 dB, S31 resonant frequency occurs at *f_1_* = 4.82 GHz with insertion loss value of −0.51 dB. The values obtained are similar to the previous device. This is relevant, because the design method provides two alternatives to build power dividers with output in different layers depending on the requirement.

**Figure 21:**
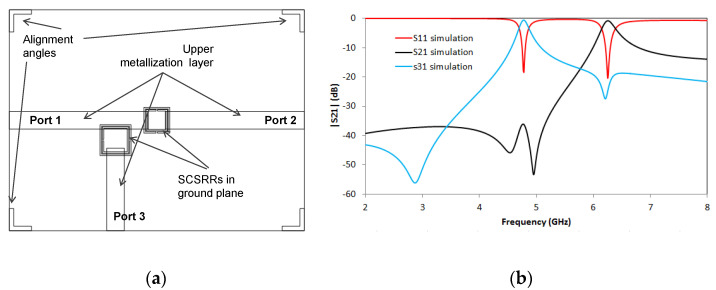
(**a**) Top view overlapped layer distribution of a 2-layer duplexer. (**b**) Simulated S11 (dashed red line) and simulated S21 (dashed black line) and simulated S31 (dashed blue line).

And to finish the set of duplexers, the next device presented is a new variant of the previous ones. In this case, both SCSRRs are placed on the ground plane, while input and output microstrip lines are located in the upper microstrip layer, as plotted in Figure 21a. Again, the ring dimensions are the same as previous devices.

The output for this device is displayed in Figure 21b and the S21 peak is obtained at *f_0_* = 6.37 GHz and a value of −0.67 dB, S31 resonant frequency occurs at *f_1_* = 4.7 GHz with an insertion loss value of −0.52 dB.

With all the previous information, a comparison of the relevant figures is shown in the following Table 4. It can be highlighted that the devices presented in the article obtained similar insertion losses and return losses to the compared one but with a significantly lower effective area λg2. As mentioned, an almost identical response when designing the outputs in different layers is obtained. The resonant frequencies are tunable, providing great flexibility in the design process.

**Figure 22:**
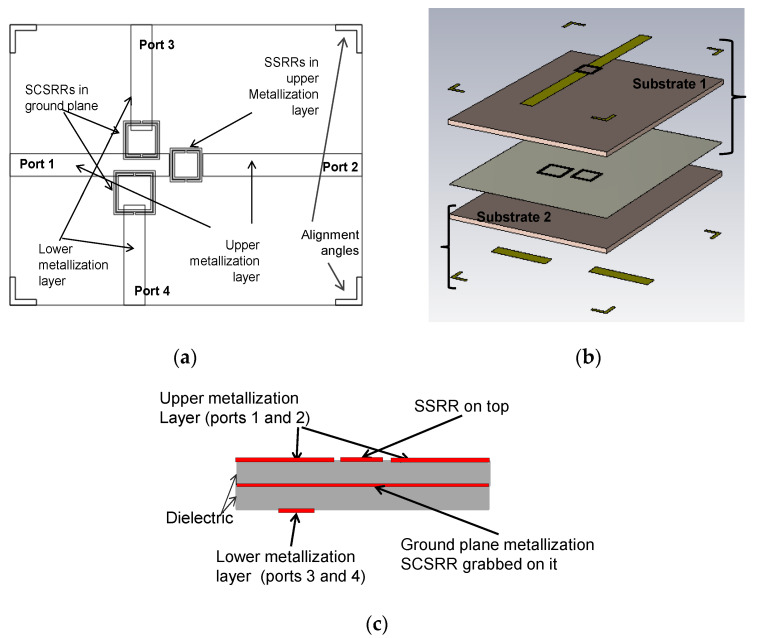
(**a**) Top view overlapped layer distribution of 2-layer triplexer, which is SSRR and SCSRR based. (**b**) Perspective view of layers composition of 2-layer triplexer, which is SSRR and SCSRR based. (**c**) Front view layer distribution.

To conclude, an evolution of presented diplexers is proposed. A collection of different triplexers will be designed in multiple configurations. They will be compared with similar implementations of multilayer triplexers [59]. The first model, proposed in this article, is designed using both kinds of resonators: SSRR and SCSRR. On the upper layer, an SSRR is used in the serial configuration. The output resonance is in Port 2 also with a microstrip line in the upper metallization layer. Two SCSRRs are obtained in the metallization layer of the ground plane just below the input line (see the layout in Figure 22a). In the lower metallization layer, output lines for Ports 3 and 4 are designed. To better understand the layer distribution in Figure 22b, a perspective layout of the layers, separated from each other, is painted. To complete the visualization, Figure 22c shows a schematic front view of the different layers with the components on them.

The relevant dimensions of the SSRR placed in the upper layer are square side length *l_2_* = 3.6 mm, the width of rings *c* = 0.2 mm, and the separation between rings *d* = 0.1 mm. For the SCSRR placed on the ground plane, closer to Port 3, the dimensions are square side length *l_3_* = 5 mm, the width of rings *c* = 0.2 mm, and the separation between rings *d* = 0.1 mm (*c* and *d* are the same for all the rings in this device). The distance between the center of the ring until the beginning of the output line to Port 3 is labeled as *p3* = 2.7 mm. For the SCSRR closer to Port 4, the square side length *l_4_* = 4.1 mm. The distance until the beginning of the output line to Port 4, *p4* = 3.3 mm. In these devices, we have also the design parameters *zi* and *di* for both resonators placed in the ground plane. To distinguish them, they will be labeled as *z_i3_* and *d_i3_* for the distances relevant to Port 3. Relevant distances to Port 4 will be named *z_i4_* and *d_i4_*. In this device, values for these dimensions are *z_i3_* = 5.05 mm, *d_i3_* = 2.65 mm, *z_i4_* = 5.85 mm, and *d_i4_* = 2.95 mm.

**Figure 23:**
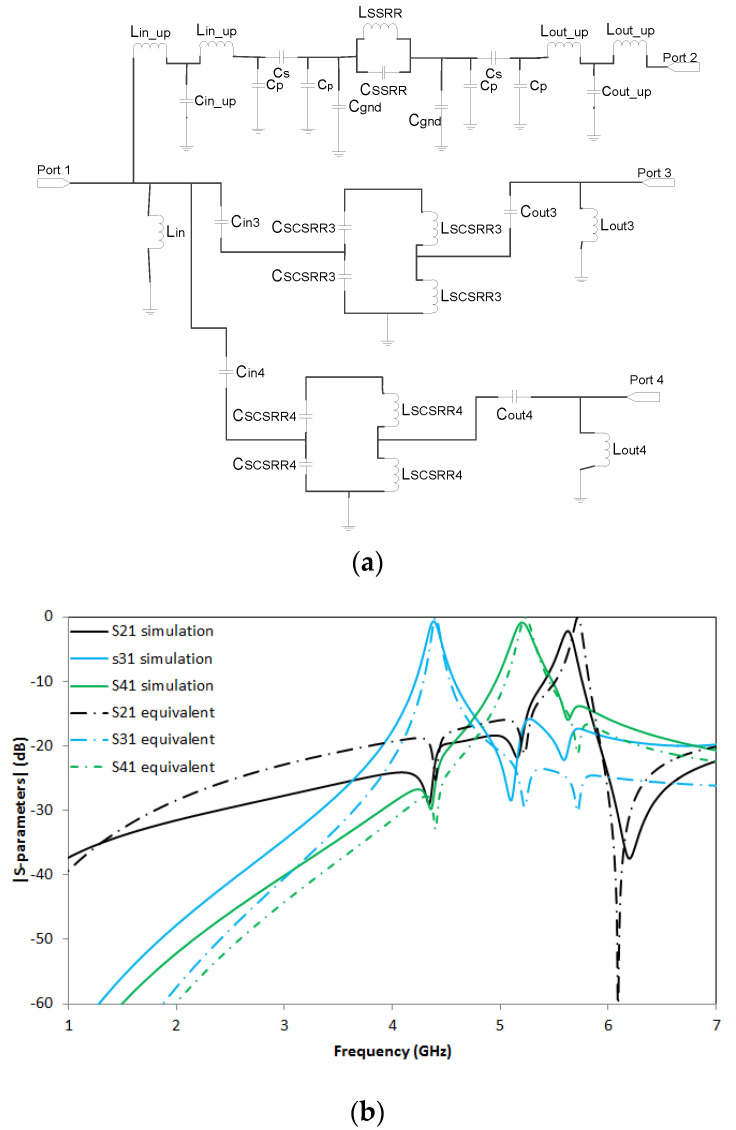
(**a**) Equivalent circuit of triplexer made with SSRR on top and two SCSRRs on ground plane. (**b**) Simulated S21 (dashed black line), equivalent circuit S21 (dashed-dotted black line), simulated S31 (dashed blue line), equivalent circuit S31 (dashed-dotted blue line), simulated S41 (dashed green line), and equivalent circuit S41 (dashed-dotted green line).

The fundamentals for the equivalent circuit of the triplexer have already been outlined. In Figure 23a, we can see three stages, namely the one above which corresponds to the upper part of the SSRR, and two paths with the SCSRRs which direct to Ports 3 and 4, respectively. The values for this circuit, are the following: *L_in_up_* = 0.01 nH, *C_in_up_* = 0.04 pF, *Cp* = 0.093 pF, *Cs* = 0.091 pF, *C_gnd_* = 0.006 pF, *C_SSRR_* = 0.39 pF, *L_SSRR_* =1.75 nH, *L_out_up_* = 0.01 nH, *C_out_up_* = 0.04 pF. *L_in_* = 3.7 nH, *C_in3_* = 0.127 pF, *C_SCSRR3_* = 1.05 pF, *L_SCSRR3_* = 1.11 nH, *C_out3_* = 0.127 pF, *L_out3_* = 5.3 nH. *C_in4_* = 0.127 pF, *C_SCSRR3_* = 0.88 pF, *L_SCSRR3_* = 0.92 nH, *C_out3_* = 0.127 pF, *L_out3_* = 5.3 nH.

The results obtained in the full wave simulation compared with the S-parameters of the equivalent circuit are depicted in Figure 23b. The resonances are obtained at *f_0_* = 5.63 GHz with insertion losses of S21 = −2.2 dB. The second resonance is *f_1_* = 4.39 GHz with a value S31= −0.73 dB, and, finally, the third resonance is *f_2_* =5.2 GHz with insertion losses of S41 = −0.86 dB.

A good response is obtained in SCSRR resonant frequencies, while SSRR exhibits lower performance. However, the relevant fact is that the three resonant frequencies are quite close to each other, with more than 18 dB of isolation between all the outputs.

**Figure 24:**
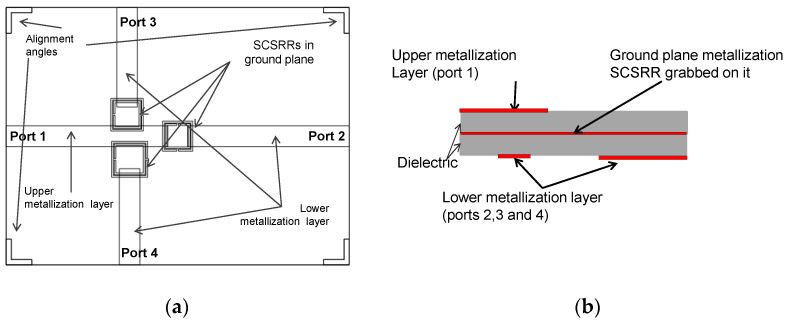
(**a**) Top view overlapped layer distribution of 2-layer triplexer SCSRR based. All output ports in upper layer are designed. (**b**) Front view layer distribution.

The following device is similar to the previous one. In this case, the SSRR has been replaced by a third SCSRR. The Source port is placed in the upper metallization layer, and the output ports are placed in the lower metallization layer. Overlapped layers are shown in Figure 24a, and the schematic representation of the layers is shown in Figure 24b. The relevant dimensions of the rings are the width of rings *c* = 0.2 mm, and the separation between rings *d* = 0.1 mm for all of them. The square side length for the SCSRR close to Port 2 is *l_2_* = 3.5 mm, for the SCSRR close to Port 3 is *l_3_* = 4.2 mm, and finally, for the SCSRR near Port 4 is *l_4_* = 3.8 mm. It has also adapted the rings’ location. Values are for SCSRR close to Port 3 and SCSRR closer to Port 4: *z_i3_* = 5.6 mm, *d_i3_* = 2.7 mm and *z_i4_* = 5.9 mm, *d_i4_* = 2.5 mm, respectively. Additionally, the parameters designed for the optimization in the output layer to attain the open stub adaptation are adjusted. In this device: *p1* = 1.15 mm, *p2* = 1.15 mm, *p3* = 1.1 mm, and *p4* = 0.9 mm.

**Figure 25:**
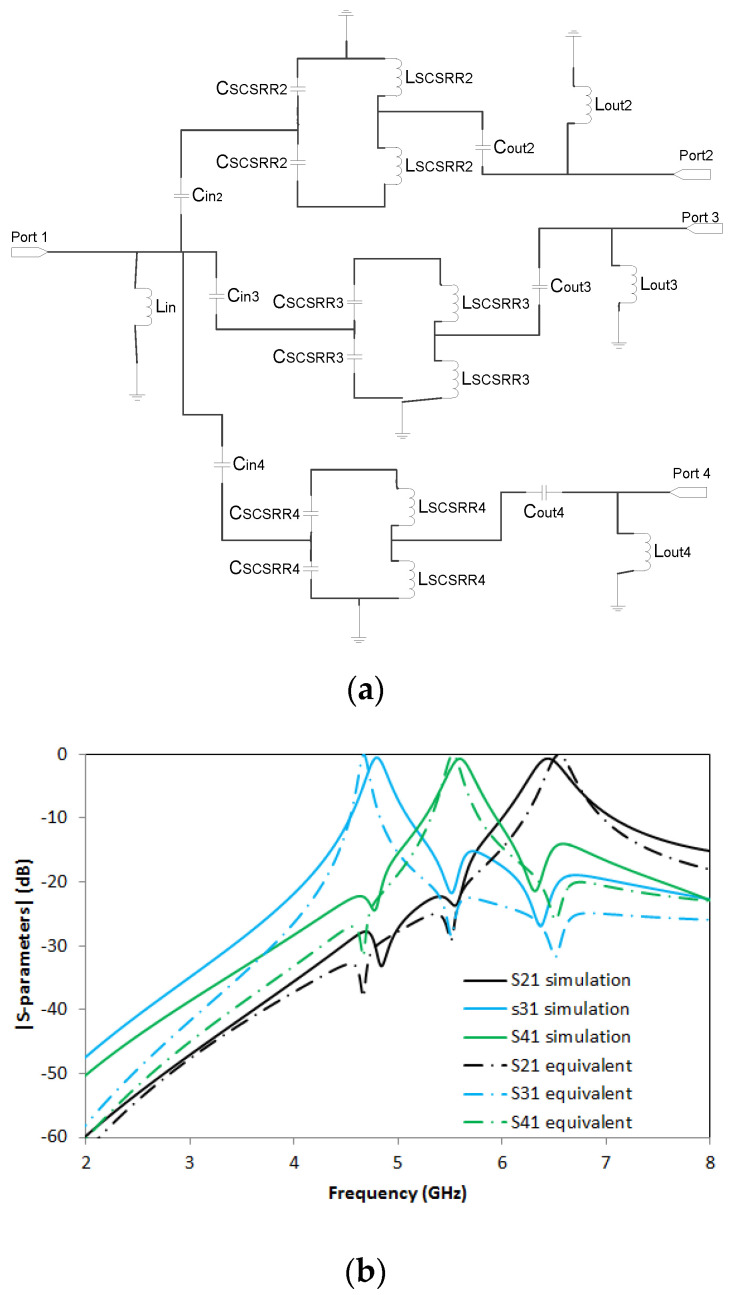
(**a**) Equivalent circuit of triplexer made with three SCSRR on ground plane. (**b**) Simulated S21 (dashed black line), equivalent circuit S21 (dashed-dotted black line), simulated S31 (dashed blue line), equivalent circuit S31 (dashed-dotted blue line), simulated S41 (dashed green line), and equivalent circuit S41 (dashed-dotted green line).

The fundamentals of the equivalent circuit for this device have also been explained in the paper. Three stages, one for each resonator, are modeled as painted in Figure 25a. The input stub is modeled with the open stub modeled with inductor L_in_, and the rest of the components are defined in the same way as the previous device.

The values for this circuit are the following: *C_in2_* = 0.127 pF, *C_SCSRR2_* = 0.69 pF, *L_SCSRR2_* = 0.73 nH, *C_out2_* = 0.127 pF, *L_out2_* = 5.3 nH. *L_in_* = 3.8 nH, *C_in3_* = 0.127 pF, *C_SCSRR3_* = 1.05 pF, *L_SCSRR3_* = 1.11 nH, *C_out3_* = 0.127 pF, *L_out3_* = 5.3 nH. *C_in4_* = 0.127 pF, *C_SCSRR3_* = 0.88 pF, *L_SCSRR3_* = 0.92 nH, *C_out3_* = 0.127 pF, and *L_out3_* = 5.3 nH. Again, the values provided for the capacitors and inductances of the resonators are the series of the LC resonators calculated in [32].

S-parameters results in comparison with equivalent circuit model results are compared in Figure 25b. Great output with less than 1 dB insertion lost in each port is achieved. The resonant frequency for the output in Port 2, *f_0_* = 6.44 GHz with a value of S21 = −0.67 dB. The resonant frequency on the resonator close to Port 3 is obtained at frequency *f_1_* = 4.79 GHz with a value of S31 = −0.53 dB, and finally, the last resonant frequency is obtained in frequency *f_2_* = 5.6 GHz with a value of S41 = −0.71 dB.

**Figure 26:**
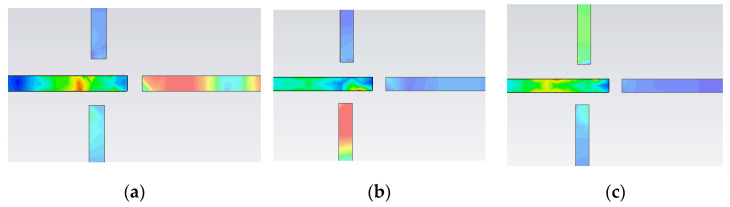
Surface current at (**a**) *f_0_* = 6.44 GHz, (**b**) *f_1_* = 4.79 GHz, and (**c**) *f_2_* = 5.6 GHz.

A good definition of the resonance peaks is obtained in all the resonant frequencies. Insertion losses are lower than 0.75 dB in the worst case, and good isolation between ports is obtained. There is good agreement between simulation and equivalent circuits. It can only be appreciated a short deviation in the frequencies, lower than 2% that can be caused by small coupling between SCSRRs not considered in our model. In Figure 26, the surface current distribution at the resonant frequencies of the different SCSRRs is displayed. Not only does most of the current flow through the designed output line but also no currents are coupled with the other ports, which makes the SCSRR transparent out of their resonant frequencies.

**Figure 27:**
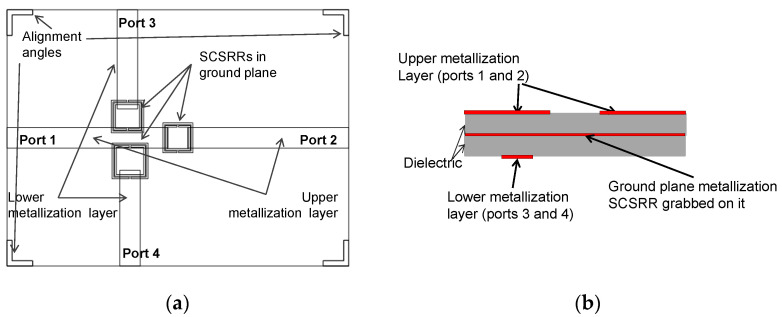

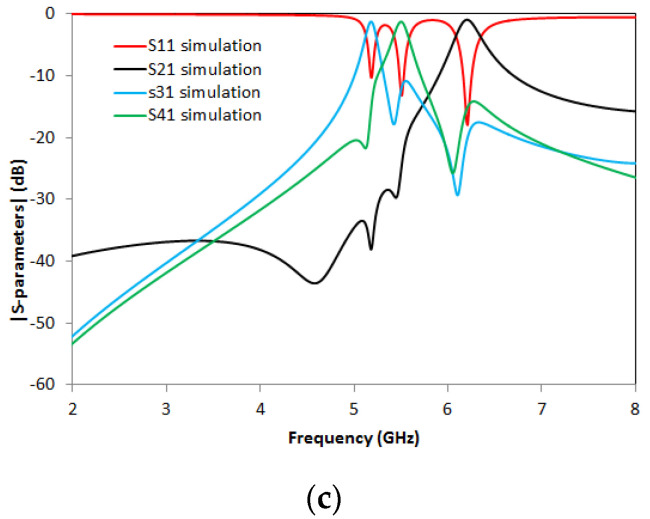
(**a**) Top view overlapped layer distribution of 2-layer triplexer, which is SCSRR based. Mixed output ports in upper and lower layer are designed. (**b**) Front view layer distribution. (**c**) Simulated S11 (dashed red line), simulated S21 (dashed black line), simulated S31 (dashed blue line), and simulated S41 (dashed green line).

For the next triplexer, again three SCSRRs are used. In this device, the output lines are designed in different layers. The output layer to Port 2 is in the upper metallization layer, that is, in the same layer as the input line. Output lines towards ports 3 and 4 are placed in the lower metallization layer. For a better understanding of the layer distribution, the top layout together with a schematic front view of the layer distribution is provided in Figure 27a,b.

Design parameter values for this device are the width of rings *c* = 0.2 mm and the separation between rings *d* = 0.1 mm, which are common in the three resonators of this device. The distance between the center of the ring until the beginning of the output line to Port 2, labeled as *p2* = 1.15 mm. The dimensions for the SCSRRs have the same dimensions as in the previous device. Only displacement parameters have been modified in order to avoid undesired couplings. For the resonator close to Port 3, the values are *zi*3 = 5.6 mm and *di*3 = 2.7 mm, and for the one close to Port 4, the values are *zi*4 = 4.9 mm and *di*4 = 2.5 mm. S-parameters values are plotted in Figure 27c.

A very good response in all three resonant frequencies is achieved, but the real advantage of this device is that the resonance frequencies are really close to each other. The ratio between the second resonance and the lower one is $f_{0d}^{2}$/$f_{0d}^{1}$ = 1.1, while the ratio between the upper frequency to the lower one is $f_{0d}^{u}$/$f_{0d}^{1}$ = 1.22.

Last but not least device, is quite similar to the previous ones but follows the same idea. In this device, all output lines are placed in the same upper metallization layer as the input line. All the ring dimensions are the same as in the previous device. There are only some changes in the location of the rings. The values are *z_i3_* = 5.6 mm and *d_i3_* = 2.7 mm, and for the SCSRR closer to the port, the values are *z_i4_* = 5.9 mm and *d_i4_* = 2.5 mm. Additionally, the parameters designed for the optimization in the output layer to attain the open stub adaptation are adjusted. In this device, *p1* = 1.15 mm, *p2* = 1.15 mm, *p3* = 1.1 mm, and *p4* = 0.9 mm are observed. 

**Figure 28:**
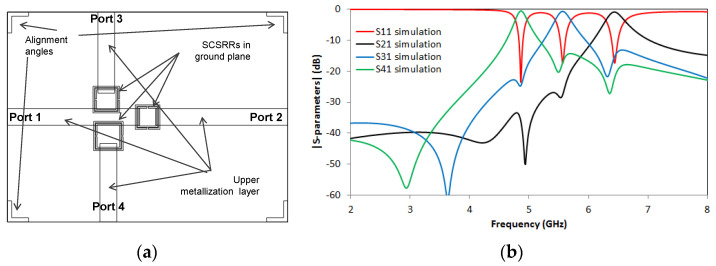
(**a**) Top view overlapped layer distribution of 2-layer triplexer, which is SCSRR based. All output ports in upper layer are designed. (**b**) Simulated S11 (red line), simulated S21 (black line), simulated S31 (blue line), and simulated S41 (green line).

In Figure 28b, the S-parameters of the previous device are shown. A great output level and more than 20 dB isolation are achieved. Additionally, the proximity of each resonance makes this device very attractive in the design of many applications.

To conclude this section, a comparison of some key figures is presented. Not only the triplexers proposed in this paper are compared but also some research on microstrip multi-layer triplexers. The comparison is presented in Table 5.

In the comparison, we can see that the proposed devices obtain similar insertion losses and return losses, with two improvements. The first one is that the effective area is much smaller, and in the second one, the ratio between resonant frequencies is lower than the compared ones. Another advantage, already demonstrated in the duplexers section, is that similar performances are achieved when the outputs are designed in different layers. 

The methodology used for diplexers and triplexers can be expanded in the design of multiplexers by simply adding more resonators and outputs, with the flexibility of building the outputs in the desired layer.

## 6. Equivalent Circuits Parameters Compilation and Summary of Main Results

This section has two objectives, namely to show the definition of the parameters and show the values of the equivalent circuits of the different devices in a grouped way. These objectives are completed to help the reader establish comparisons easily. The second objective presents a summary of the best results obtained from the different devices.

Starting with the first, Table 6 lists all the equivalent circuits with their associated parameters and their values. For those equivalent circuits with more than one component with a similar name in the picture, all the components will be listed in the same cell. 

To finalize this section, a new Table 7 is provided with all the results obtained in the new devices proposed in the paper. In the table, the most relevant figures will be highlighted considering that one of the main advantages of the multilayer has nothing to do with the metrics but has everything to do with the flexibility provided in the design of outputs in different layers.

## 7. Conclusions

A complete characterization of the Complementary Split Ring Resonator in a multi-layer environment is developed in this article. High performance in miniaturized devices is presented and studied. Single-layer, double-layer, double-frequency resonators, power dividers, duplexers, and triplexers are analyzed, exhibiting multi-frequency resonances with well-defined port responses. A sample of all possible combinations is shown, but all others would be valid, that is, a combination of SSRR, CSSRR, and outputs in the same layer or different layers. The implementation is in three output devices, but the proposed designs can be increased by adding more rings along the input line. The proposed methodology and the corresponding devices can be readily adapted for their integration into transceivers and radiating systems for current wireless communication systems and future developments in 6G systems in the millimeter wave range and beyond. Future work is foreseen in relation to the experimental validation of the proposed prototypes with adequate fixtures in the frequency ranges of the operation of devices.

## Figures and Tables

**Table 1:** Schema of the structure of this article.

CSRR as Elementary Particle		
**Resonators** Serial SCSRR Second layer output Double frequency Perpendicular output	**Power dividers** Single layer Outputs on top Outputs on down Mixed outputs	**Duplexers** SSRR + SCSRR Outputs top and down Outputs on top layer Outputs on lower layer **Triplexers** SSRR + SCSRR Outputs mixed layer Outputs on top layer Outputs on lower layer

**Table 2:** Comparison between different models of resonators.

Ref	f_0_ (GHz)	Effect Size λ_g_^2^	Insertion Loss (dB)	Return Loss (dB)
[25]	5	0.005	0	21
[39]	1.95	0.021	3	20
This work (Figure 4)	5.01	0.0019	0.91	18.8
This work (Figure 5)	4.24	0.0027	1.1/1.2	15.4/19.3
This work (Figure 8)	5	0.0019	0.83	22.1
This work (Figure 10)	4.74	0.0021	0.71	22

**Table 3:** Comparison between different models of power dividers.

Reference	f_0_ (GHz)	Effect Size λ_g_^2^	Insertion Loss (dB)	Efficiency (%)
[21]	2.45	0.0019	4.25/4.7	85
[40]	6.3	0.0012	N/A	N/A
[47]	5.00	0.0019	3.8/4	90
This work (Figure 11)	5.24	0.0017	3.24/4.29	75.5
This work (Figure 13)	5.63	0.0019	4.03/4.69	85.9
This work (Figure 15)	4.72	0.0019	3.67/3.70	99
This work (Figure 16)	5.00	0.0021	3.53/3.89	90.5

**Table 4:** Comparison between different models of diplexers.

Reference	Effect Size λ_g_^2^	$f_{0d}^{1,u}$ (GHz)	$f_{0d}^{u}$ $/f_{0d}^{1}$	$\mathrm{IL}(\mathrm{dB})\;f_{0d}^{1,u}$	$\mathrm{RL}(\mathrm{dB})\;f_{0d}^{1,u}$
[55]	0.218	2.49/2.98	1.19	1.15/1.54	35/33
[56]	0.185	2.45/3.6	1.47	1.45/1.3	24/19
[57]	0.0089	1/2	2	N/A	38.8/48.9
This work (Figure 17)	0.055	4/5.57	1.39	0.79/2.2	27.3/12.9
This work (Figure 19)	0.0025	4.8/6.4	1.33	0.53/0.52	17.2/29
This work (Figure 20)	0.0024	4.7/6.1	1.3	0.51/0.71	20/22
This work (Figure 21)	0.0028	3.98/5.4	1.35	0.52/0.67	19/20

**Table 5:** Comparison between different models of triplexers.

Reference	Effect Size λ_g_^2^	$f_{0d}^{1,2,u}$ (GHz)	$f_{0d}^{2}$ $/f_{0d}^{1}$ $f_{0d}^{u}$ $/f_{0d}^{1}$	$\mathrm{IL}(\mathrm{dB})\;f_{0d}^{1,2,u}$	$\mathrm{RL}(\mathrm{dB})\;f_{0d}^{1,2,u}$
[54]		1/2/2.4	2/2.4	0.9/1.8/2.4	>10.8
[59]	0.075	0.8/1/1.2	1.25/1.5	2.2	>10
This work (Figure 22)	0.0021	4.4/5.1/5.5	1.16/1.25	0.7/0.9/2.2	25.9/21.9/16.1
This work (Figure 24)	0.0030	4.9/5.5/6.5	1.12/1.32	0.56/0.71/0.66	21.8/15.9/23.9
This work (Figure 27)	0.0028	5.1/5.6/6.2	1.10/1.22	1.2/1.8/0.9	10.2/13.1/17.7
This work (Figure 28)	0.0026	5/5.4/6.4	1.08/1.28	0.54/0.68/0.91	21.1/17.2/14.8

**Table 6:** Equivalent circuits, parameters, and values.

Device	Fig.	L_in_ (nH)	C_in_ (pF)	C_SCSRR_ _(pF)_	L_SCSRR_ (nH)	L_out_ (nH)	C_out_ (pF)	L_SSRR_ (nH)	C_SSRR_ (pF)	C_p_ (pF)	C_s_ (pF)	C_gnd_ (pF)
Resonator. SCSRR in microstrip	(Figure 1c)	1.16 ^1^	0.296 ^1^	0.380	1.690	-	-	-	-	-	-	-
Resonator. Serial resonator SCSRR 1 layer	(Figure 4a)	1.16	0.296	0.81 ^2^	0.87 ^3^	1.16	0.296	-	-	-	-	-
Resonator. Double frequency	(Figure 5b)	1.1	0.24	1.12	1	1.1	0.24	7.1	0.062	0.009	0.091	0.006
Resonator. Serial resonator SCSRR 2 layers	(Figure 8a)	1.16	0.296	0.81 ^2^	0.87 ^3^	1.16	0.296	-	-	-	-	-
Resonator. SCSRR 2 layer-cross outputs	(Figure 4a) ^4^	1.16	0.296	0.81 ^2^	0.87 ^3^	1.16	0.137	-	-	-	-	-
Power divider. Cross output both 2nd layer	(Figure 14a)	1.16	0.296	0.81 ^2^	0.87 ^3^	1.16 1.16	0.19 0.19	-	-	-	-	-
Power divider. Serial + cross Outputs both 2nd layer	(Figure 16b)	1.16	0.296	0.81 ^2^	0.87 ^3^	1.16 1.16	0.137 0.296	-	-	-	-	-
Duplexer. SSRR + SCSRR. Outputs in two layers	(Figure 18a)	0.01 ^5^ 2.2	0.04 ^5^ 0.24	1.08	1.19	0.01 ^5^ 1.6	0.04 ^5^ 0.24	7.1	0.062	0.009	0.091	0.006
Duplexer 2 SCSRRs All outputs bottom layer	(Figure 19b)	3.8	0.102 0.102	0.69 0.99	0.74 1.06	5.3 5.3	0.102 0.102	-	-	-	-	-
Triplexer. SSRR + 2 SCSRRs	(Figure 23a)	0.01 3.7	0.04 0.127 0.127	1.05 0.88	1.11 0.92	0.01 5.3 5.3	0.01 0.127 0.127	1.75	0.39	0.009	0.091	0.006
Triplexer. 3 SCSRRs All outputs bottom layer	(Figure 25a)	3.8	0.127 0.127 0.127	0.69 1.05 0.88	0.73 1.11 0.92	5.3 5.3 5.3	0.127 0.127 0.127	-	-	-	-	-

^1^ In Figure 1c, the elements are called L_T_ and C_T_. ^2^ To consider this, there are two capacitors in serial configuration. ^3^ To consider this, there are two inductors in serial configuration. ^4^ It contains the same components, so the pictures are the same with different values. ^5^ The first value corresponds to C_xx_up_ or L_xx_up_ and the second C_in_, C_out_, L_in,_ and Lout, respectively.

**Table 7:** Main figure proposed devices.

Device	Fig.	$f_{0d}^{1,u}$ (GHz)	Effect Size λg2	Insertion Loss (dB)	Return Loss (dB)	Efficiency (%)	$f_{0d}^{u}$ $/f_{0d}^{1}$
Resonator. SCSRR in microstrip	(Figure 1d)	4.71	-	38	-	-	-
Resonator. Serial resonator SCSRR 1 layer	(Figure 4b)	5.01	0.0019	0.91	18.8	-	-
Resonator. Double frequency	(Figure 5b)	4.24 5.51	0.0027	1.1 1.2	15.4 19.3	-	-
Resonator. Serial resonator SCSRR 2 layers	(Figure 8b)	5	0.0019	0.83	22.1	-	-
Resonator. SCSRR 2 layer-cross outputs	(Figure 10)	4.74	0.0021	0.71	22	-	-
Power divider All outputs on the top layer	(Figure 12)	5.24	0.0017	3.24 4.29	-	75.5	-
Power divider. Cross output both 2nd layer	(Figure 14b)	5.63	0.0019	4.03 4.69	-	85.9	-
Power divider. Serial + cross Outputs in different layers	(Figure 15b)	4.72	0.0019	3.67 3.70	-	99	-
Power divider. Serial + cross Outputs both 2nd layer	(Figure 16c)	5.00	0.0021	3.53 3.89	-	90.5	-
Duplexer. SSRR + SCSRR. Outputs in different layers	(Figure 18b)	4 5.57	0.055	0.79 2.2	27.3 12.9	-	1.39
Duplexer 2 SCSRRs All outputs bottom layer	(Figure 19c)	4.8 6.4	0.0025	0.53 0.52	17.2 29	-	1.33
Duplexer 2 SCSRRs Outputs in different layers	(Figure 20b)	4.7 6.1	0.0024	0.51 0.71	20 22	-	1.3
Duplexer 2 SCSRRs All outputs on the top layer	(Figure 21b)	3.98 5.4	0.0028	0.52 0.67	19 20	-	1.35
Triplexer SSRR + 2 SCSRRs Outputs in different layers	(Figure 23b)	4.4 5.1 5.5	0.0021	0.7 0.9 2.2	25.9 21.9 16.1	-	1.16 1.25
Triplexer 3 SCSRRs All outputs bottom layer	(Figure 25a)	4.9 5.5 6.5	0.0030	0.56 0.71 0.66	21.8 15.9 23.9	-	1.12 1.32
Triplexer 3 SCSRRs Outputs in different layers	(27c)	5.1 5.6 6.2	0.0028	1.2 1.8 0.9	10.2 13.1 17.7	-	1.10 1.22
Triplexer 3 SCSRRs All outputs on the top layer	(Figure 28b)	5 5.4 6.4	0.0026	0.54 0.68 0.91	21.1 17.2 14.8	-	1.08 1.28

## References

[1] Tataria H., Shafi M., Molisch A.F., Dohler M., Sjöland H., Tufvesson F. (2021). 6G Wireless Systems: Vision, Requirements, Challenges, Insights, and Opportunities. Proc. IEEE.

[2] Popovski P., Chiariotti F., Huang K., Kalor A.E., Kountouris M., Pappas N., Soret B. (2022). A Perspective on Time Toward Wireless 6G. Proc. IEEE.

[3] Shojaeifard A., Wong K.-K., Tong K.-F., Chu Z., Mourad A., Haghighat A., Hemadeh I., Nguyen N.T., Tapio V., Juntti M. (2022). MIMO Evolution Beyond 5G Through Reconfigurable Intelligent Surfaces and Fluid Antenna Systems. Proc. IEEE.

[4] Ding Z., Lv L., Fang F., Dobre O.A., Karagiannidis G.K., Al-Dhahir N., Schober R., Poor H.V. (2022). A State-of-the-Art Survey on Reconfigurable Intelligent Surface-Assisted Non-Orthogonal Multiple Access Networks. Proc. IEEE.

[5] Al-Bawri S.S., Islam M.T., Islam S., Singh M.J., Alsaif H. (2022). Massive metamaterial system-loaded MIMO antenna array for 5G base stations. Sci. Rep..

[6] Shabbir T., Islam M.T., Al-Bawri S.S., Aldhaheri R.W., Alharbi K.H., Aljohani A.J., Saleem R. (2020). 16-Port Non-Planar MIMO Antenna System With Near-Zero-Index (NZI) Metamaterial Decoupling Structure for 5G Applications. IEEE Access.

[7] Bin Ashraf F., Alam T., Islam M.T. (2018). A Uniplanar Left-Handed Metamaterial for Terrestrial Microwave Links. IEEE Microw. Wirel. Components Lett..

[8] Lerosey G., Fink M. (2022). Wavefront Shaping for Wireless Communications in Complex Media: From Time Reversal to Reconfigurable Intelligent Surfaces. Proc. IEEE.

[9] Martini E., Maci S. (2022). Theory, Analysis, and Design of Metasurfaces for Smart Radio Environments. Proc. IEEE.

[10] Gupta K.C., Bahl I., Garg R. (1979). Microstrip Lines and Slotlines.

[11] Vegesna S., Saed M.A. (2012). Compact two-layer microstrip bandpass filters using broadside-coupled resonators. Prog. Electromagn. Res. B.

[12] Ho C.-H., Fan L., Chang K. Slot-coupled double-sided microstrip interconnects and couplers. Proceedings of the 1993 IEEE MTT-S International Microwave Symposium Digest.

[13] Jaisson D. (2000). Multilayer microstrip directional coupler with discrete coupling. IEEE Trans. Microw. Theory Tech..

[14] Herscovici N., Pozar D. (1991). Full-wave analysis of aperture-coupled microstrip lines. IEEE Trans. Microw. Theory Tech..

[15] Omar A., Dib N. (1997). Analysis of slot-coupled transitions from microstrip-to-microstrip and microstrip-to-waveguides. IEEE Trans. Microw. Theory Tech..

[16] Huang X., Wu K.-L. (2012). A Broadband and Vialess Vertical Microstrip-to-Microstrip Transition. IEEE Trans. Microw. Theory Tech..

[17] Yang L., Zhu L., Choi W.-W., Tam K.-W. Wideband vertical microstrip-to-microstrip transition designed with cross-coupled microstrip/slotline resonators. Proceedings of the 2015 Asia-Pacific Microwave Conference (APMC).

[18] Tanaka T., Tsunoda K., Aikawa M. (1988). Slot-coupled directional couplers between double-sided substrate microstrip lines and their applications. IEEE Trans. Microw. Theory Tech..

[19] Schwab W., Menzel W. (1992). On the design of planar microwave components using multilayer structures. IEEE Trans. Microw. Theory Tech..

[20] Meissner P., Kitlinski M. (2005). A 3-dB multilayer coupler with UC-PBG structure. IEEE Microw. Wirel. Components Lett..

[21] You S.-J., Liao W.-J. A multi-layer coupled-line power divider. Proceedings of the 2008 8th International Symposium on Antennas, Propagation and EM Theory.

[22] Falcone F., Lopetegi T., Baena J., Marques R., Martin F., Sorolla M. (2004). Effective negative-/spl epsiv/stopband microstrip lines based on complementary split ring resonators. IEEE Microw. Wirel. Components Lett..

[23] Pendry J.B., Holden A.J., Robbins D.J., Stewart W.J. (1999). Magnetism from conductors and enhanced nonlinear phenomena. IEEE Trans. Microw. Theory Tech..

[24] Falcone F., Lopetegi T., Laso M., Baena J.D., Bonache J., Beruete M., Marqués R., Martin F., Sorolla M. (2004). Babinet Principle Applied to the Design of Metasurfaces and Metamaterials. Phys. Rev. Lett..

[25] Hashemi S.K. Dual-band bandpass filters based on multilayer ring resonators. Proceedings of the 2011 11th Mediterranean Microwave Symposium (MMS).

[26] Bonache J., Gil I., Garcia-Garcia J., Martin F. (2006). Novel microstrip bandpass filters based on complementary split-ring resonators. IEEE Trans. Microw. Theory Tech..

[27] Mondal P., Mandal M., Chaktabarty A., Sanyal S. (2006). Compact bandpass filters with wide controllable fractional bandwidth. IEEE Microw. Wirel. Components Lett..

[28] Gil M., Bonache J., Garcia-Garcia J., Martel J., Martin F. (2007). Composite Right/Left-Handed Metamaterial Transmission Lines Based on Complementary Split-Rings Resonators and Their Applications to Very Wideband and Compact Filter Design. IEEE Trans. Microw. Theory Tech..

[29] Jarauta E., Laso M.A.G., Lopetegi T., Falcone F., Beruete M., Baena J.D., Marcotegui A., Bonache J., García J., Marqués R. (2006). Novel microstrip backward coupler with metamaterial cells for fully planar fabrication techniques. Microw. Opt. Technol. Lett..

[30] Horestani A.K., Abbott D., Fumeaux C. (2013). Rotation Sensor Based on Horn-Shaped Split Ring Resonator. IEEE Sens. J..

[31] Zamora G., Zuffanelli S., Aguila P., Paredes F., Martin F., Bonache J. (2018). Broadband UHF-RFID Passive Tag Based on Split-Ring Resonator (SRR) and T-Match Network. IEEE Antennas Wirel. Propag. Lett..

[32] Baena J., Bonache J., Martin F., Sillero R., Falcone F., Lopetegi T., Laso M., Garcia-Garcia J., Gil I., Portillo M. (2005). Equivalent-circuit models for split-ring resonators and complementary split-ring resonators coupled to planar transmission lines. IEEE Trans. Microw. Theory Tech..

[33] Aznar F., Bonache J., Martín F. (2008). Improved circuit model for left-handed lines loaded with split ring resonators. Appl. Phys. Lett..

[34] Naqui J., Duran-Sindreu M., Martin F. (2013). Modeling Split-Ring Resonator (SRR) and Complementary Split-Ring Resonator (CSRR) Loaded Transmission Lines Exhibiting Cross-Polarization Effects. IEEE Antennas Wirel. Propag. Lett..

[35] Liu J.C., Lin H.C., Zeng B.H., Yeh K.D., Chang D.C. (2010). An Improved Equivalent Circuit Model for CSRR-Based Bandpass Filter Design With Even and Odd Modes. IEEE Microw. Wirel. Compon. Lett..

[36] Pozar D.M. (2012). Microwave Engineering.

[37] Jarauta E., Falcone F., Beruete M. (2014). High-Q series coupled microstrip split-ring resonator device. Waves Random Complex Media.

[38] Marqués R., Medina F., Rafii-El-Idrissi R. (2002). Role of bianisotropy in negative permeability and left-handed metamaterials. Phys. Rev. B.

[39] Boonlom K., Kerdsumang S., Akkaraekthalin P. A miniaturized bandpass filter using two-layers of microstrip open-loop resonators. Proceedings of the 2005 13th IEEE International Conference on Networks Jointly Held with the 2005 IEEE 7th Malaysia International Conf on Communic.

[40] Hashemi S.K., Mirmohammadi S.N., Dalasm Z.K. Balun and power divider based on multilayer ring resonators (MRR). Proceedings of the 2017 IEEE 17th International Conference on Ubiquitous Wireless Broadband (ICUWB).

[41] Boonlom K., Pratumvinit T., Akkaraekthalin P. (2009). A compact microstrip two-layers bandpass filter using improved interdigital-loop resonators. Proceedings of the 2009 IEEE International Symposium on Radio-Frequency Integration Technology (RFIT).

[42] Choudhary D.K., Chaudhary R.K. A compact SIW based filtering power divider with improved selectivity using CSRR. Proceedings of the 2017 Progress in Electromagnetics Research Symposium—Fall (PIERS—FALL).

[43] Danaeian M., Moznebi A., Afrooz K., Hakimi H. (2016). Miniaturised equal/unequal SIW power divider with bandpass response loaded by CSRRs. Electron. Lett..

[44] Shen X., Feng W., Chen H., Che W. Narrowband Filtering Balun Power Divider Based on SIW and CSRRs. Proceedings of the 2018 International Conference on Microwave and Millimeter Wave Technology (ICMMT).

[45] Jibreel O., Dib N., Shamaileh K.A. Miniaturized Bailey Power Divider Using SRRs. Proceedings of the 2018 IEEE International Symposium on Antennas and Propagation USNC/URSI National Radio Science Meeting.

[46] Yang X., Hong D.H. A 4-GHz 1:10 unequal Wilkinson power divider using split ring resonator. Proceedings of the 2017 3rd IEEE International Conference on Computer and Communications (ICCC).

[47] Li Q., Gong J., Shi X., Wang X., Wei F. A compact broadband multi-layer in-phase power divider. Proceedings of the 2010 International Conference on Microwave and Millimeter Wave Technology.

[48] Inoue N., Kawakami T., Horii Y., Kitamura T. A super-compact dual-band Wilkinson power divider composed of multi-layered CRLH transmission lines. Proceedings of the 40th European Microwave Conference.

[49] Zeng H.Y., Wang G.M., Wei D.Z., Wang Y.W. (2012). Planar diplexer using composite right-/ left-handed transmission line under balanced condition. Electron. Lett..

[50] An J., Wang G., Zhang C., Zhang P. (2018). Diplexer using composite right-/left-handed transmission line. Electron. Lett..

[51] Bonache J., Gil I., Garcia-Garcia J., Martin F. (2005). Complementary split ring resonators for microstrip diplexer design. Electron. Lett..

[52] García-Lampérez A., Gómez-García R., Salazar-Palma M. Compact diplexer with edge-coupled and nonbianisotropic split-ring resonators. Proceedings of the 2012 IEEE/MTT-S International Microwave Symposium Digest.

[53] Huang Y., Wen G., Li J. Compact and highly-selective microstrip bandpass filter and diplexer using two-stage twist modified split-ring resonators. Proceedings of the 2015 IEEE MTT-S International Microwave Symposium.

[54] Tang C.-W., You S.-F. (2006). Design methodologies of LTCC bandpass filters, diplexer, and triplexer with transmission zeros. IEEE Trans. Microw. Theory Tech..

[55] Yang T., Chi P., Itoh T. (2011). Compact Quarter-Wave Resonator and Its Applications to Miniaturized Diplexer and Triplexer. IEEE Trans. Microw. Theory Tech..

[56] Fernández-Prieto A., Lujambio A., Martel J., Medina F., Martín F., Boix R.R. (2018). Balanced-to-Balanced Microstrip Diplexer Based on Magnetically Coupled Resonators. IEEE Access.

[57] Radonić V., Crnojević-Bengin V., Baskakova A., Vendik I. Multilayer microwave diplexers based on dual-mode resonators for ISM/WiFi bands. Proceedings of the 2014 Mediterranean Microwave Symposium (MMS2014).

[58] Horii Y., Caloz C., Itoh T. (2005). Super-compact multilayered left-handed transmission line and diplexer application. IEEE Trans. Microw. Theory Tech..

[59] Aksoy S.C., Yıldı İ. A multilayered triplexer based on interdigital filter topology with PCB technology. Proceedings of the 2015 IEEE MTT-S International Microwave Symposium.

